# Altered duodenal N6-methyladenosine levels in common variable immunodeficiency associate with duodenal microbiota

**DOI:** 10.3389/fimmu.2026.1875823

**Published:** 2026-07-08

**Authors:** Vegard Myhre, Mari Kaarbø, Mingyi Yang, Børre Fevang, Mirta M. L. Sousa, Henrik M. Reims, Knut E. A. Lundin, Johannes R. Hov, Pål Aukrust, Magnar Bjørås, Silje F. Jørgensen

**Affiliations:** 1 Division of Surgery and Specialized Medicine, Research Institute of Internal Medicine, Oslo University Hospital, Oslo, Norway; 2 Institute of Clinical Medicine, University of Oslo, Oslo, Norway; 3 Department of Microbiology, Oslo University Hospital and University of Oslo, Oslo, Norway; 4 Department of Medical Biochemistry, Oslo University Hospital, Oslo, Norway; 5 Section of Clinical Immunology and Infectious Diseases, Oslo University Hospital, Rikshospitalet, Oslo, Norway; 6 Department of Clinical and Molecular Medicine, Norwegian University of Science and Technology, NTNU, Trondheim, Norway; 7 Proteomics and Modomics Experimental Core Facility (PROMEC) at Norwegian University of Science and Technology, Trondheim, Norway; 8 Department of Pathology, Oslo University Hospital, Rikshospitalet, Oslo, Norway; 9 Section of Gastroenterology, Department of Transplantation Medicine, Oslo University Hospital, Rikshospitalet, Oslo, Norway; 10 Norwegian PSC Research Center, Department of Transplantation Medicine, Oslo University Hospital, Oslo, Norway

**Keywords:** CVID - common variable immunodeficiency, duodenum, epitranscriptome analysis, gastrointestinal diseases, M6A modification, m6A enzyme system, microbiota, primary immunodeficiencies (PID)

## Abstract

**Introduction:**

Common variable immunodeficiency (CVID) is frequently complicated by duodenal inflammation, but the underlying molecular mechanisms remain poorly understood. While epigenetic alterations have been described in CVID, the epitranscriptome is largely unexplored. We therefore investigated whether RNA N6-methyladenosine (m6A) modifications in duodenal tissue are altered in CVID and whether such changes are associated with the local microbiota or m6A-related enzymes.

**Methods:**

m6A modification levels were analysed in snap-frozen duodenal biopsies from CVID patients with intraepithelial lymphocytosis and inflammation (CVID_IEL; n = 5), CVID patients with normal duodenal histology (CVID_N; n = 5) and controls with normal biopsies (n = 5) using m6A-RNA immunoprecipitation followed by microarray profiling and gene set enrichment analysis. Duodenal bacterial microbiota from the same anatomical region were characterised by 16S ribosomal RNA gene sequencing, and selected m6A-regulating enzymes were quantified in biopsies by targeted proteomics.

**Results:**

In total, 4,134 differentially methylated transcripts were identified, and unsupervised principal component analyses revealed partially overlapping, but clearly divergent m6A signatures for CVID_IEL, CVID_N and controls, with a gradient along the first principal component. Pathway analysis showed relative hypermethylation of mitochondria- and ribosome-related gene sets in both CVID subgroups versus controls, and hypomethylation of pathways linked to ubiquitination, proteasomal degradation, glycosylation and post-transcriptional gene silencing in CVID_IEL versus CVID_N. Sparse canonical correlation models demonstrated significant associations between specific duodenal bacterial genera and m6A-modified transcripts in CVID, but not in controls, whereas expression levels of the examined m6A-regulating enzymes did not differ between groups.

**Discussion:**

These findings suggest that duodenal inflammation in CVID may be associated with a distinct m6A epitranscriptomic signature that is linked to specific features of the mucosal microbiota, providing preliminary, hypothesis-generating evidence for a potential interaction between microbiota, epitranscriptomic regulation and local immune dysregulation in CVID.

## Introduction

Common variable immunodeficiency (CVID) is the most common symptomatic primary immunodeficiency in adults, with a prevalence of approximately 1 in 25,000-50,000 (1, 2). CVID is characterised by a B-cell defect with impaired immunoglobulin (Ig) production, leading to recurrent respiratory infections, particularly with encapsulated bacteria. In addition, up to 70% of the patients develop inflammatory and autoimmune complications that frequently involve the gastrointestinal (GI) tract (3). The most consistent histological finding in the upper GI tract is increased intraepithelial lymphocytes (IEL) in the duodenum, which histologically resembles celiac disease. However, transcriptomic and epigenomic profiling of duodenal tissue suggests that this inflammation is more likely driven by viral triggers and/or dysregulated immune responses to the gut microbiota rather than gluten (4–6).

Beyond classical epigenetic mechanisms, where environmental triggers alter gene expression through DNA modifications, gene regulation is also shaped post-transcriptionally by epitranscriptomic modifications of RNA. Such modifications influence mRNA stability, decay, splicing and translation, thereby modulating cell differentiation and functional responses including immune and inflammatory responses (7–10). Among these marks, N6-methyladenosine (m6A) is the most abundant internal modification in eukaryotic mRNA and is also present in several non-coding RNA (9, 11, 12).

Dysregulation of m6A has been implicated in cancer, autoimmunity, and inflammatory bowel disease (13–16), conditions that are also overrepresented in CVID (2, 4, 17, 18). Moreover, both disturbed gut microbial composition and persistent immune activation, key features in subgroups of CVID patients (3, 19, 20), have been shown in experimental models to influence m6A deposition and function (21, 22). In line with this, gut microbial colonisation has been shown to substantially alter the intestinal m6A epitranscriptome in mice (23, 24), and pathobionts such as *Fusobacterium nucleatum* and enterotoxigenic *Bacteroides fragilis* can modulate METTL3/METTL14-dependent m6A-methylation in colonic tissue in experimental models, thereby promoting inflammation and tumour progression (25, 26). Furthermore, m6A regulators such as METTL3 have been reported to be important for intestinal epithelial regeneration and immune homeostasis in experimental colitis, supporting a potential role of m6A signalling in intestinal inflammation (27).

However, to the best of our knowledge, m6A RNA modifications have not previously been characterised in CVID. In the present study, we hypothesised that dysregulated m6A RNA modifications are associated with duodenal inflammation in CVID patients and are linked to the disturbed gut microbiota previously reported in these patients. To test this, we analysed m6A modification levels in duodenal biopsies from CVID patients with and without duodenal inflammation, as well as from controls. We further examined whether m6A patterns correlated with the local mucosal microbiota and with the expression of m6A-regulating enzymes in the same tissue.

## Materials and methods

### Study design

At the time of inclusion, CVID was defined according to international diagnostic criteria as decreased serum levels of IgG, IgA and/or IgM (at least two standard deviations below the age-adjusted mean), exclusion of other causes of hypogammaglobulinemia (28).

All CVID patients were recruited between 2012–2013 at the Section of Clinical Immunology and Infectious Diseases, Oslo University Hospital, Rikshospitalet, Oslo, Norway. Exclusion criteria were acute infection, acute exacerbation of inflammatory/autoimmune condition or treatment with immunomodulatory therapy. The controls were recruited from individuals that were referred to the same endoscopy unit as the patients. Prior to inclusion as a control, referrals were assessed by KEAL and SFJ for eligible candidates. Individuals with a low suspicion of GI disease, and with no other relevant medical history, were invited by letter to participate in the study a few weeks before their planned endoscopy. The reasons for the referral were typically abdominal pain or suspicion of gastroesophageal reflux. If the histological description of the duodenal biopsy was described as normal by the pathologist, they were included as controls. Biopsies from the proximal duodenum were collected during upper endoscopy (GIFHQ190, Olympus, Hamburg, Germany) according to protocol at the Section for Gastroenterological Endoscopy at Oslo University Hospital, Rikshospitalet, Oslo, Norway (4).

For the present study, duodenal biopsy samples from CVID patients were primarily sub-grouped according to duodenal histology into those with increased intraepithelial lymphocytes (IELs) and inflammation (CVID_IEL) and those with normal duodenal mucosa (CVID_N). Increased IELs in the duodenal mucosa were defined as ≥25 IELs per 100 epithelial cells, in line with guideline-based thresholds (29, 30). All biopsies used for m6A, microbiota and proteomics analyses were snap-frozen and stored in liquid nitrogen for subsequent extraction of RNA, DNA and protein. Extended methodological details are provided in the **Supplementary Information**.

CVID patients were further clinically characterised according to the presence or absence of non-infectious complications, using previously published criteria (see **Supplementary Methods**).

Routine clinical genetic testing for monogenic CVID-like disorders was not implemented at the time of inclusion, and no patients had undergone such analyses. After the later introduction of a targeted next-generation sequencing panel for primary immunodeficiencies in clinical practice, all 10 CVID patients were retrospectively tested using this panel (598 genes associated with primary immunodeficiency and haematological diseases; https://www.genetikkportalen.no/find-ngs/1100, version 4).

### Ethical approval

The study was approved by the Regional Committee for Medical and Health Research Ethics (REC, reference: 33256) and conducted in accordance with the Declaration of Helsinki. Biopsies were collected in the general biobank “Tarmsykdommer” (REC: 20521). Written informed consent was obtained from all participants.

### m6A-RNA immunoprecipitation (m6A-RIP) and microarray analyses

Total RNA was isolated from duodenal biopsy samples using the All-Prep DNA/RNA/protein kit (Qiagen, Hilden, Germany) according to the manufacturer’s instructions with minor modifications as previously described (6). m6A RNA immunoprecipitation (me-RIP) and microarray analysis were performed by Arraystar (Rockville, MD, USA) using their Human m6A-mRNA&lncRNA Epitranscriptomic microarray platform (protocol at available at: https://www.arraystar.com/epitranscriptomic-array-service-m6a/m5c/m1a/ac4c/m7g/-/). The complete workflow protocol is described in detail in the **Supplementary Information**.

### Quantification of enzymes involved in m6A RNA regulation using targeted mass spectrometry

Quantification of m6A RNA enzymes were performed using targeted mass spectrometry (MS) coupled with liquid chromatography (LC). LC-MS/MS, as described in more detail in the **Supplementary Information**. Protein pellets were obtained from duodenal biopsy samples using the AllPrep DNA/RNA/Protein All Prep kit, as above (6). A targeted proteomic panel including 12 m6A-regulating enzymes and four additional RNA editing enzymes was quantified by LC MS/MS; all targeted proteins and detection status are listed in **Supplementary Table 1**.

### Bacterial microbiota analysis in duodenum

Bacterial DNA was extracted using an established protocol and subjected to high-throughput amplicon sequencing of the 16S rRNA gene (31). Taxonomic classification of amplicon sequence variants was performed in Qiime2 using a naive Bayes classifier trained on the V3-V4 region of a pre-clustered version (99% similarity) version of Silva database v138 as previously described (6).

### Statistical analysis

All statistical analyses were performed in R (v4.2.3). The complete workflow is described in detail in the **Supplementary Information**.

Differentially m6A methylated transcripts (DMTs) between groups were identified using moderated t-tests with Benjamini-Hochberg adjustment for multiple testing. Transcripts were considered differentially methylated if they fulfilled both of the following criteria: adjusted p-value p_adj_ < 0.05 and an absolute difference in mean methylation level greater than 15% between groups (∣Δmethylation| > 0.15).

## Results

### Clinical characteristics

**Figure 1:**
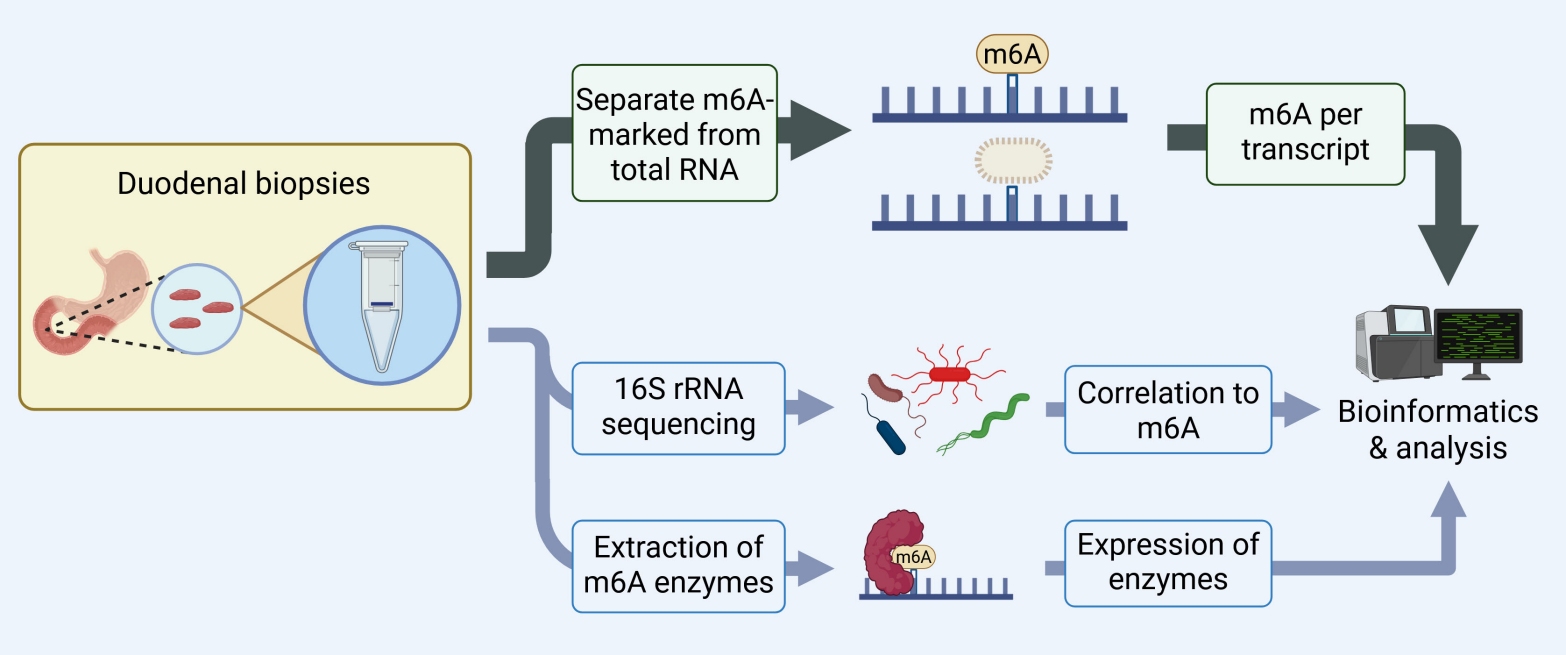
Overall workflow. In this study, we analysed the expression of the N6-methyladenosine (m6A) RNA modification in duodenal biopsies from CVID compared to controls (top, green). Additional analysis (bottom, light blue) included investigating m6A correlations to the duodenal microbiota and measuring the expression of m6A related enzymes in biopsies. Figure made using BioRender. CVID, common variable immunodeficiency; m6A, N6-methyladenosine.

A simplified overview of the study is provided in **Figure 1**. For the m6A analyses, we included duodenal biopsies from five CVID_IEL patients, five CVID_N patients and five controls with normal histology. Clinical characteristics are summarised in **Table 1**. All CVID patients were on immunoglobulin replacement therapy as part of standard care (six patients on subcutaneous immunoglobulin and four patients on intravenous immunoglobulin). Of note, none of the CVID patients were on probiotic supplement or receiving prophylaxis antibiotics at the time of inclusion. There were no significant demographic or clinical differences between the CVID subgroups, except for chronic diarrhoea, which was present in all CVID_IEL patients, and in one CVID_N patient.

**Table 1:** Characteristics of the m6A study cohort.

Characteristic	CVID_N	CVID_IEL	Controls	P-value
**Number of patients, n**	5	5	5	–
**Female, n**	3	1	2	0.74
**Age (years), mean(min-max)**	43 (28-61)	43 (35-58)	49 (32-67)	0.30
**Infection only**	1	0		1.00
Complications				
*Autoimmune cytopenia, n*	1	1	–	1.00
Chronic diarrhoea*, n	1	5	–	0.05
*Lymphoid-hyperplasia, n*	3	4	–	1.00
*Organ specific autoimmunity, n*	0	1	–	1.00
*Splenomegaly, n*	2	4	–	0.52
*Autoimmunity, n*	1	2	–	1.00
Immunoglobulin therapy				
*IVIG, n*	2	2	–	–
*SCIG, n*	3	3	–	–
**Monogenic variant, n**	2^†^	1^‡^	–	–

CVID_IEL refers to CVID patients with increased intraepithelial lymphocytes and inflammation in the duodenum, while CVID_N refers to CVID patients without this increase and inflammation. P-values were calculated using Fisher’s exact, except in age were Kruskal-Wallis was used. *Chronic diarrhoea was defined as diarrhoea lasting more than three months, based on a GI symptom questionnaire (Gastrointestinal Symptom Rating Scale-Irritable Bowel Syndrome, GSRS-IBS) and exclusion of GI infection. †IKZF1, NFKB2 ‡TNFAIP3.

Genetic testing was performed retrospectively using a targeted next-generation sequencing panel for primary immunodeficiencies. Monogenic variants compatible with a CVID phenotype were identified in three of ten patients (TNFAIP3, IKZF1 and NFKB2). Only the patient with the TNFAIP3 variant had duodenal inflammation (CVID_IEL), whereas the patients with IKZF1 and NFKB2 variants had normal duodenal histology (CVID_N; **Table 1**). No pathogenic or likely pathogenic variants were identified in the remaining patients.

### m6A expression levels in duodenal biopsies distinguish CVID from controls

**Figure 2:**
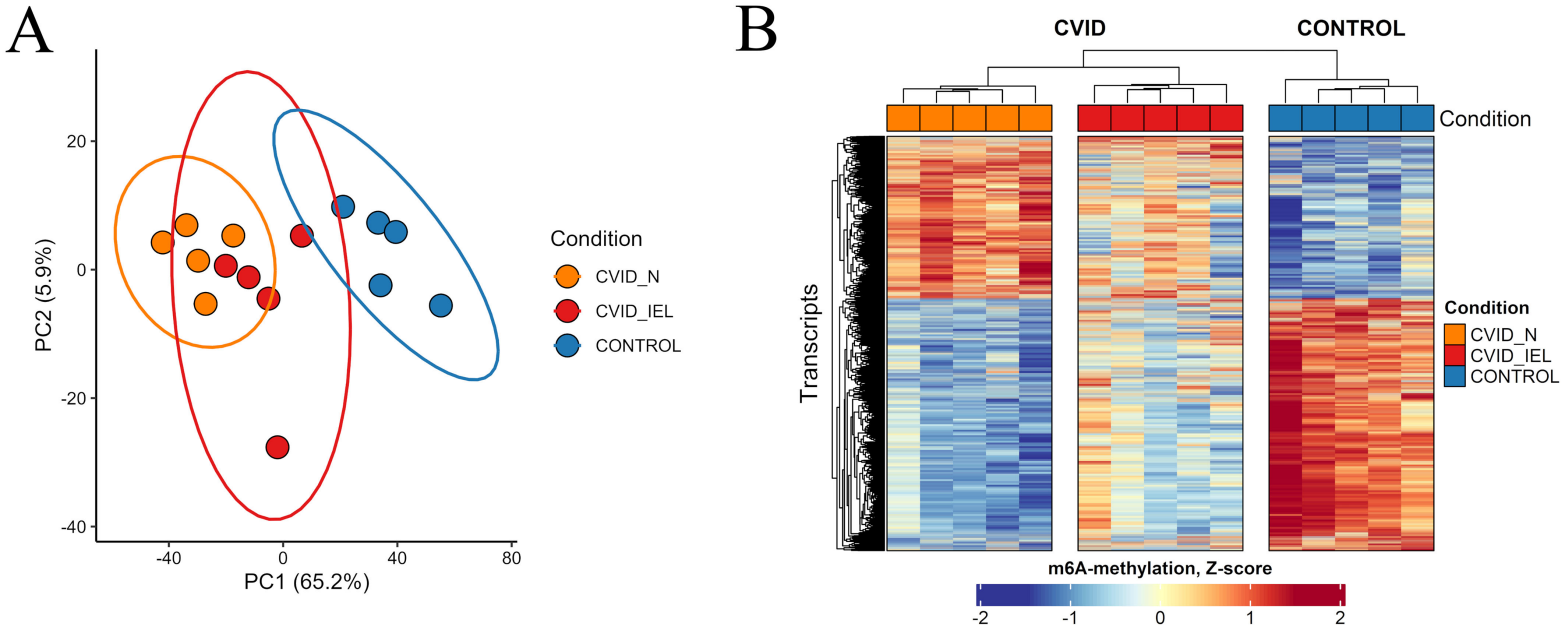
Unsupervised clustering of differentially methylated transcripts. **(A)** Principal component analysis of the 4,134 DMTs in the main cohort. Ellipses represent 95% confidence interval within a condition, based on the principal component 1 (PC1) and 2 (PC2) coordinates. **(B)** Heatmap of the 4,134 DMTs. Top dendrogram show clustering of conditions then samples, left dendrogram show clustering of DMTs. CVID, common variable immunodeficiency; CVID_IEL, CVID with increased intraepithelial lymphocytes and inflammation in the duodenum; CVID_N, CVID patients with normal duodenum.

We first compared all CVID patients to controls. In the principal component analysis (PCA), CVID samples separated largely from controls (**Figure 2A**). Comparing CVID to controls, we identified 4,134 differentially methylated transcripts (DMTs) by moderated t-test with Benjamini-Hochberg adjustment, applying an adjusted p-value threshold of p_adj_ < 0.05 and an absolute mean methylation difference greater than 15% (**Supplementary Excel File 1**). A hierarchically clustered heatmap of these DMTs, with samples grouped by clinical category and clustered within each group, showed a clear separation between CVID and control biopsies based on their m6A profiles (**Figure 2B**). CVID_N samples displayed methylation patterns that were largely opposite to those of controls, whereas CVID_IEL samples exhibited a distinct pattern that differed from both CVID_N and controls. For comparison, a fully unsupervised version of the heatmap, in which sample order is determined solely by m6A-based clustering without constraining group order, is provided in **Supplementary Figure 1**.

### Altered m6A expression in CVID subgroups with and without inflammation compared to controls

**Figure 3:**
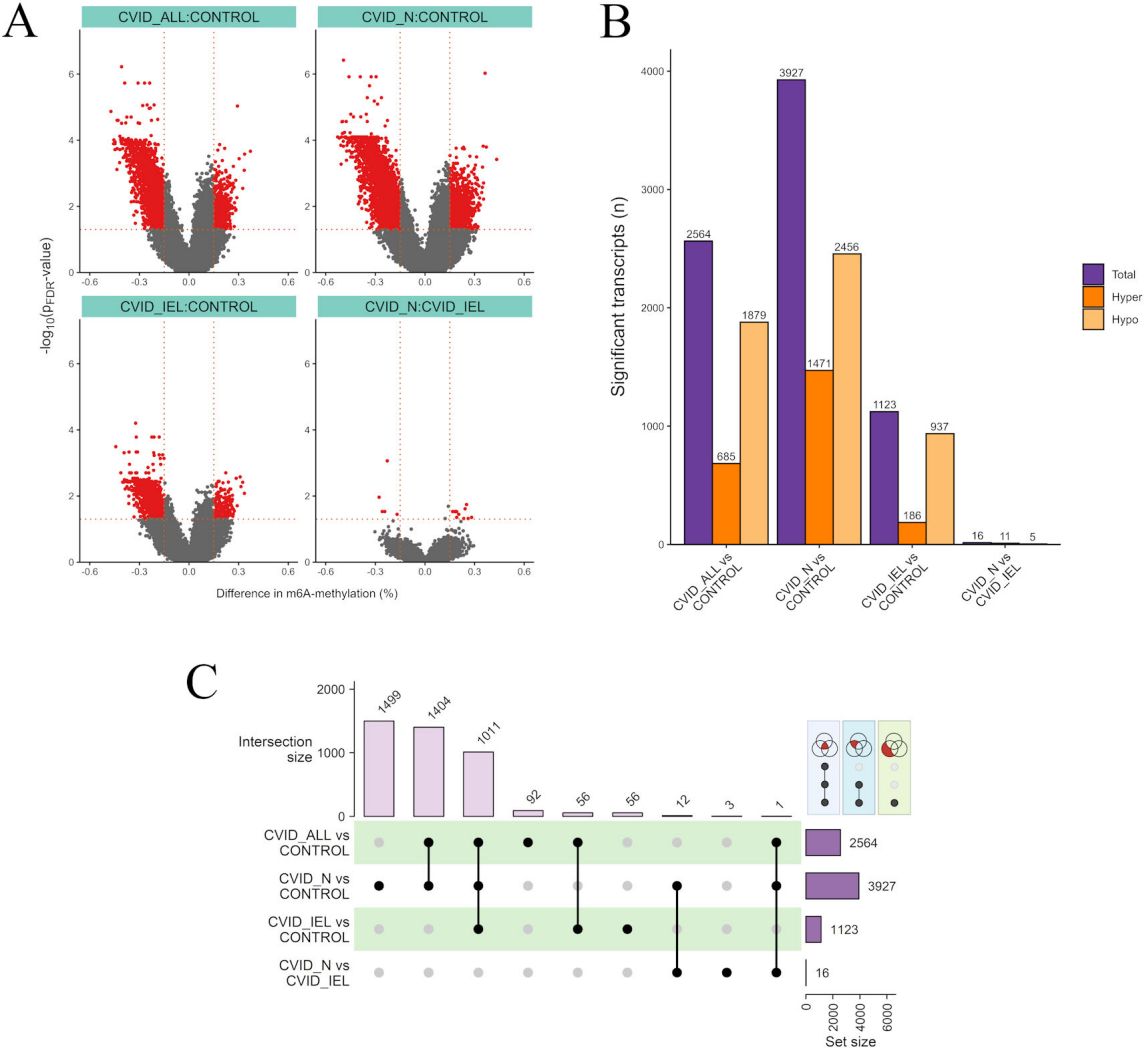
Distribution of differentially methylated transcripts (DMTs). **(A)** Vulcanoplots for the differential comparisons. **(B)** Overall distribution of total, hyper- and hypomethylated transcripts. **(C)** UpSet-plot showing the distribution of all DMTs and their overlap across the main CVID-comparisons. The Venn-diagram (top right) illustrates how different overlap areas correspond to dots and connecting lines in an UpSet-plot. CVID, common variable immunodeficiency; CVID_IEL, CVID with increased intraepithelial lymphocytes and inflammation in the duodenum; CVID_N, CVID patients with normal duodenum; CVID_ALL, CVID_IEL and CVID_N merged into one group.

We next examined m6A patterns in CVID subgroups defined by duodenal histology. CVID with intraepithelial lymphocytosis (CVID_IEL) and CVID with normal duodenal histology (CVID_N) showed a partial separation in the PCA, with CVID_IEL samples generally shifting towards higher PC1 values and CVID_N towards lower PC1 values, although the 95% confidence ellipses overlapped. Notably, one CVID_N sample clustered together with the CVID_IEL group in the heatmap (**Figures 2A,B**). We identified 1,123 DMTs for CVID_IEL versus controls and 3,927 DMTs for CVID_N versus controls (**Figures 3A,B**). **Figure 3B** summarises the overall distribution of total, hyper- and hypomethylated transcripts in each comparison, showing a predominance of hypomethylated transcripts in CVID_ALL versus controls and in both CVID subgroups versus controls. The distribution of hyper- and hypomethylated transcripts across RNA biotypes is shown in **Supplementary Figure 2**.

Direct comparison of CVID_IEL and CVID_N yielded 16 DMTs, including 11 protein coding mRNAs (four hypomethylated and seven hypermethylated; **Figure 3B**, **Supplementary Figure 2**). Although the number of DMTs between subgroups was limited, several of these transcripts have potential immunological relevance, such as AGO4 (RNA mediated gene silencing) (32), ITCH (E3 ubiquitin ligase involved in immune regulation) (33, 34) and TFC7L2 (Wnt signalling and MYC regulation) (35, 36) (**Supplementary Table 2**). Overlap and subgroup specific DMTs across all comparisons are illustrated in **Figure 3C**.

### Epitranscriptomic enrichment analysis suggests dysregulation of ubiquitin, proteasome and post-transcriptional gene silencing of RNA pathways in CVID patients with duodenal inflammation

**Figure 4:**
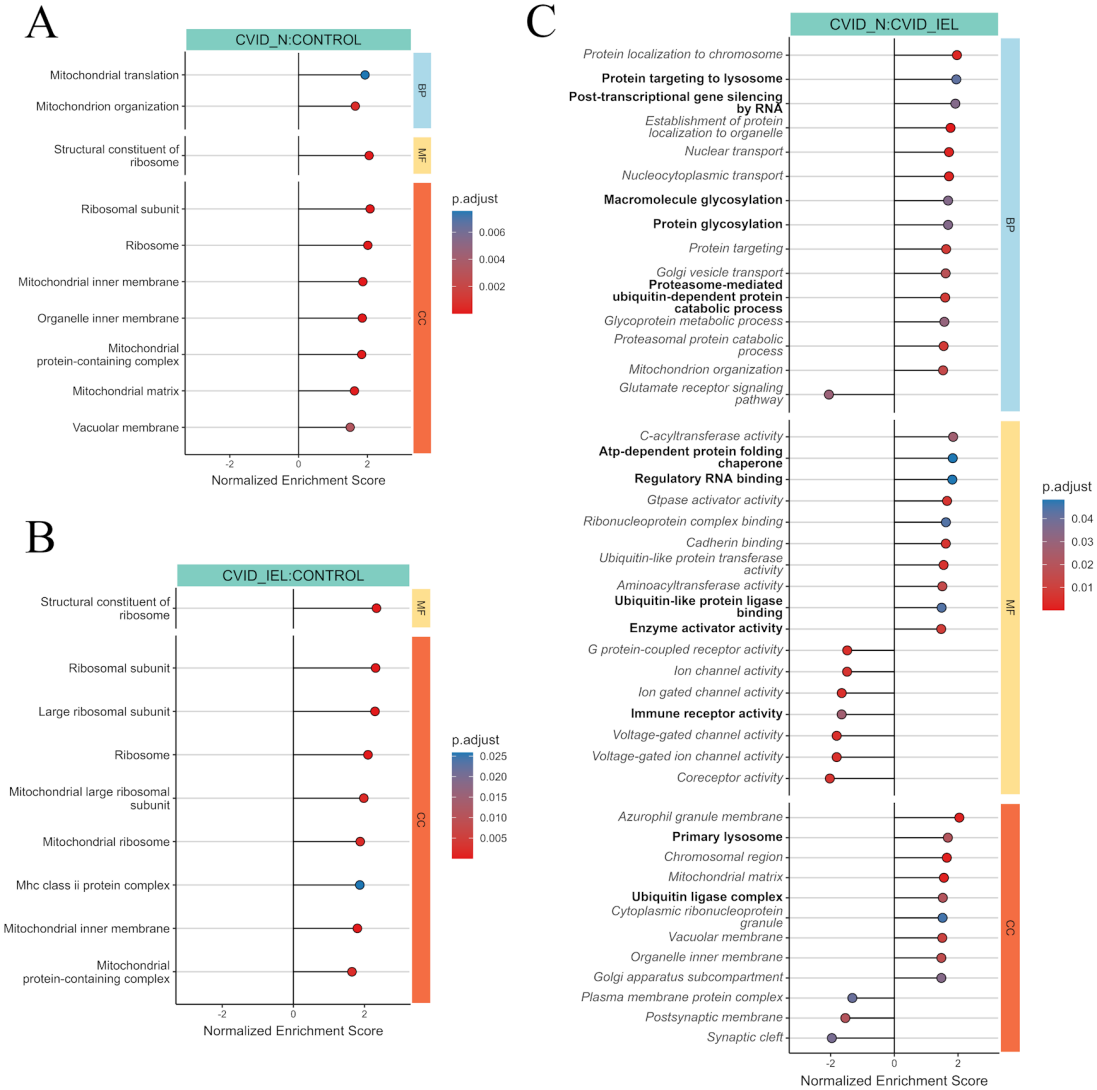
Gene set enrichment analysis of m6A in differential comparisons. The Gene Ontology (GO) databased was used, split into its three subcategories; biological process (BP), molecular function (MF), and cellular component (CC). A higher absolute normalised enrichment score (NES) indicates a stronger enrichment, while a positive/negative NES indicates relative hyper- or hypomethylation within a comparison. **(A)** Top 10 enriched gene sets (sorted by smallest adjusted p-value) for CVID_N versus controls. **(B)** Top 10 enriched gene sets (sorted by smallest adjusted p-value) for CVID_IEL versus controls. **(C)** All enriched gene sets in the comparison CVID_N versus CVID_IEL, with enrichments we found particular interesting marked in bold. CVID, common variable immunodeficiency; CVID_IEL, CVID with increased intraepithelial lymphocytes and inflammation in the duodenum; CVID_N, CVID patients with normal duodenum.

Gene set enrichment analysis of m6A levels in mRNA revealed enrichment of gene sets related to mitochondrial function and ribosomal components in both CVID-subgroups compared to controls (**Figures 4A,B**, **Supplementary Excel File 2**). These gene sets showed positive normalised enrichment scores, indicating relative hypermethylation. In addition, CVID_IEL showed hypermethylation of gene sets related to major histocompatibility complex class II (MHC II) protein complexes compared to controls (**Figure 4B**).

Although the number of individual DMTs between CVID_IEL and CVID_N was modest, pathway-level analysis revealed several regulatory processes differentially enriched between the subgroups (**Figure 4C**). These included pathways linked to ubiquitination and the proteasome, molecular chaperones, glycosylation and post-transcriptional gene silencing by RNA, all of which were relatively hypomethylated in CVID_IEL. Together, these patterns point towards altered epitranscriptomic regulation of protein homeostasis and RNA silencing pathways in inflamed duodenal mucosa in CVID.

### Correlation of m6A methylation levels with gut bacterial microbiota in CVID patients with duodenal inflammation

We next explored whether duodenal m6A patterns were associated with the local bacterial microbiota. Paired m6A and 16S rRNA sequencing data from the same anatomical region were available in twelve individuals (CVID_IEL n=4, CVID_N n=4, controls n=4) (**Supplementary Table 3**).

**Figure 5:**
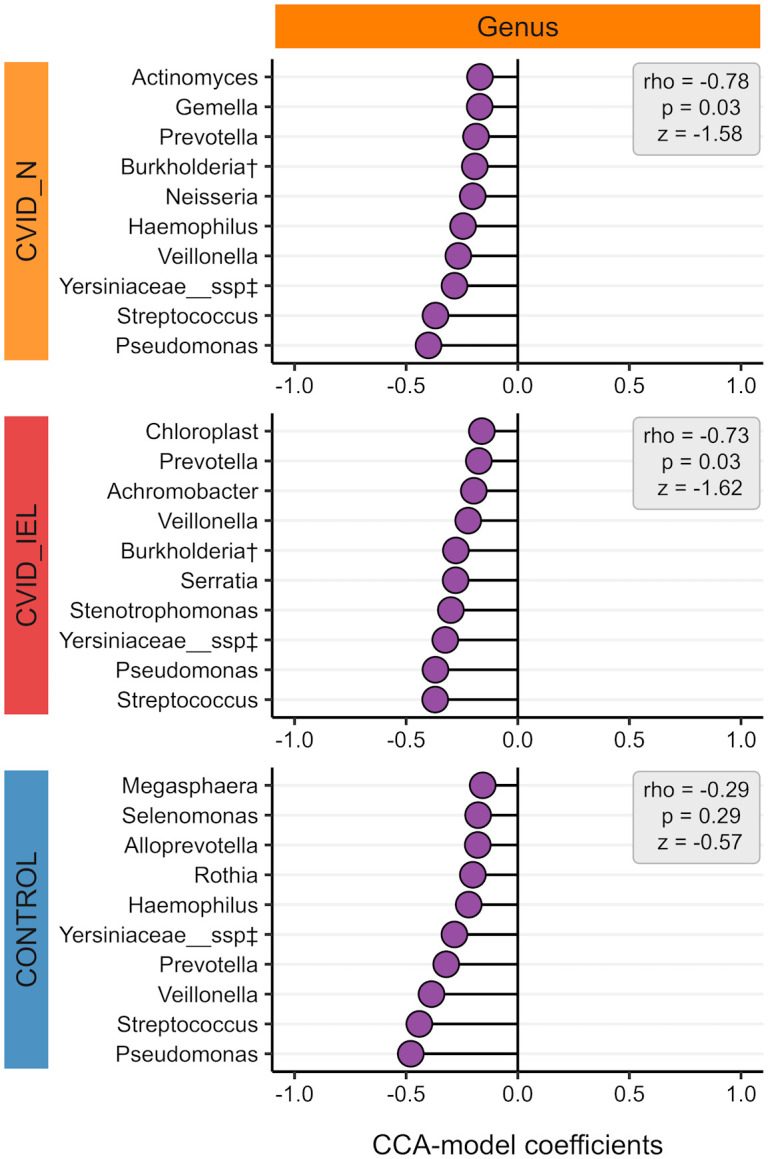
Correlation of m6A with microbiota. Top 10 bacteria genera that contribute the most to the sparse canonical correlation models. The higher the absolute value of the coefficients, the more impact the bacteria have on the model. Rho show Pearson correlation for the model. P-values and z-scores were calculated using permutation. †=The group Burkholderia-Caballeronia-Parabukholderia was shortened to Burkholderia for space considerations. ‡=Some higher taxonomic groups were included in a lower taxon in the annotation, and here assumed to be a collection of bacteria within the higher taxon that fell outside the lower taxon’s annotations (i.e. a collection group). CVID, common variable immunodeficiency; CVID_IEL, CVID with increased intraepithelial lymphocytes and inflammation in the duodenum; CVID_N, CVID patients with normal duodenum.

Alpha-diversity metrics (Faith’s PD, observed features, Pielou’s evenness and Shannon entropy) tended to be lower in CVID_IEL than in CVID_N, although these differences did not reach statistical significance (**Supplementary Figure 3A**). There were no significant correlations between global measures of microbial alpha-diversity measurements and median m6A- methylation across all transcripts at the sample level in CVID subgroups or controls (**Supplementary Figure 3B**). We therefore constructed sparse canonical correlation models using individual transcripts and bacterial taxa to identify more specific associations. Permutation testing revealed significant inverse correlations between sets of bacterial genera and m6A modified transcripts in CVID_IEL and CVID_N, but notably, not in controls (**Supplementary Excel File 3**). The ten genera with the highest contributions to the model are shown in **Figure 5** (additional taxa in **Supplementary Figure 4**). In CVID_IEL, these included potential pathogens such as Streptococcus, Pseudomonas, Yersiniaceae, Serratia and Burkholderia, as well as genera previously linked to inflammatory conditions such as Prevotella and Veillonella.

### Targeted analyses of enzymes involved in m6A RNA regulation showed no differences between conditions

To examine whether altered m6A methylation could be explained by differences in the expression of m6A regulating enzymes, we quantified a panel of 12 m6A related and four RNA editing enzymes by targeted proteomics in an available subset of duodenal biopsies (CVID_IEL n=11, CVID_N n=9, controls n=10; **Supplementary Tables 1**, **4**). Nine enzymes were reliably detected. None showed significant differences in expression between CVID and controls or between CVID subgroups (**Supplementary Figure 5**; **Supplementary Excel File 4**), indicating that the observed m6A differences are not accompanied by major changes in the protein abundance of these measured enzymes.

### m6A RNA methylation partially reflects protein expression patterns in CVID duodenal tissue

We previously reported shot gun proteomic profiles from the same cohort (6). To explore potential functional links between m6A methylation and protein expression, we matched m6A modified transcripts to quantified proteins (4,101 proteins in total, corresponding to approximately 25% of methylated mRNAs that could be annotated to protein-coding genes (see **Supplementary Methods**). Thus, 75% of the m6A modified transcripts were not captured at the protein level in this dataset.

Using moderated t-tests, however, we identified one protein (TXNDC5), an endoplasmic reticulum (ER)-resident protein involved in protein folding, particularly during hypoxia (37), that showed significant differences at both the m6A and protein levels in CVID_IEL versus controls. In CVID_N versus controls, four proteins (GCHFR, SLC25A10, DBNL, IGLL5), implicated in mitochondrial regulation, B cell pathology, macrophage function and phagocytosis (38–41), were significantly altered at both levels (**Supplementary Excel File 5**). The low number of proteins with concordant changes at the m6A and protein levels is likely influenced by the restricted coverage of methylated mRNAs at the protein level, the modest sample size, stringent multiple-testing correction, and the complexity of post-translation modification.

## Discussion

To the best of our knowledge, this is the first study to explore epitranscriptomic alterations in the duodenum in CVID. We report three main novel observations. First, duodenal tissue from CVID patients shows widespread m6A methylation changes and displays distinct, pathway level m6A signatures compared to controls, with hypermethylation of mitochondria and ribosome related gene sets. Second, the two CVID subgroups are not identical: CVID_IEL shows additional epitranscriptomic changes, including hypomethylation of pathways linked to ubiquitination and the proteasome, as well as post transcriptional gene silencing in inflamed duodenal mucosa. Third, duodenal m6A patterns form distinct correlation networks with specific mucosal bacterial taxa in CVID, but not in controls. Taken together, these findings are compatible with previously unrecognised epitranscriptomic mechanisms in the CVID gut that may contribute to local immune dysregulation and that appear to be linked, at least in part, to disturbed mucosal microbiota.

CVID pathogenesis is thought to arise from a complex interplay between genetic susceptibility and environmental factors (3, 17). We have previously shown that epigenetic DNA methylation is altered in duodenal tissue from CVID patients (5), and others have highlighted mitochondrial dysfunction and aberrant immune signalling in peripheral blood cells (42–45). The present data extend these observations to the epitranscriptomic level, and suggest that m6A-mediated regulation of RNA metabolism in the gut may be part of the immunopathological landscape in CVID.

The enrichment of mitochondria- and ribosome- related gene sets among hypermethylated transcripts in both CVID subgroups is intriguing, given the central role of these organelles in immunometabolism and effector function (43–45). Mitochondria shape innate and adaptive immune responses through regulation of reactive oxygen species, apoptosis and inflammasome activation (44–46). Our findings raise the possibility that m6A-dependent regulation of mitochondrial and ribosomal genes contributes to the altered immune activation observed in the CVID gut, although functional studies are required to establish causality.

At the subgroup level, pathway analysis suggested that CVID_IEL is characterised by hypomethylation of transcripts involved in ubiquitination and proteasomal degradation, molecular chaperones, glycosylation and post transcriptional gene silencing. These processes are central to antigen processing, cytokine signalling, lymphocyte activation and maintenance of tolerance (33, 34, 47–51). The identification of ITCH and AGO4 among the DMTs between CVID_IEL and CVID_N is intriguing, given their established roles in ubiquitin mediated protein turnover and RNA- silencing pathways (32–34, 51) and suggests that post-transcriptional regulation of these genes may contribute to CVID-associated gut inflammation. Although the limited sample size precludes firm conclusions, these patterns, including the involvement of ITCH and AGO4, are consistent with disrupted control of protein homeostasis and immune regulation in inflamed duodenal mucosa. Moreover, we identified TXNDC5, a protein involved in protein folding, particularly during hypoxia, with significant differences between CVID_IEL and controls at both the m6A and protein levels (37). Together, with the enrichment of pathways related to protein folding and degradation, this dual alteration of TXNDC5 supports a role for perturbed proteostasis in CVID associated duodenal inflammation.

The observed associations between m6A patterns and specific duodenal bacterial genera in CVID are compatible with a potential mechanistic link between the mucosal microbiota and the epitranscriptome. Previous work in animal models has demonstrated that gut bacteria can modulate m6A deposition in intestinal and hepatic tissues, and that m6A in turn can influence barrier integrity and immune responses (23, 52, 53). In our cohort, genera contributing most strongly to the m6A-microbiota correlation models were mainly Gammaproteobacteria, such as Pseudomonas, Serratia, Yersiniaceae and Burkholderia, a group that is enriched in dysbiotic faecal microbiota and associated with systemic immune activation in CVID (20, 54) and increased in duodenal mucosa in CVID enteropathy (6). We also identified Streptococcus, Prevotella and Veillonella, with Veillonella being enriched in faecal dysbiosis in CVID in independent cohorts (54, 55), and these taxa have been linked to dysbiosis and mucosal inflammation in other chronic inflammatory and immune mediated conditions (56–58). Many of these genera are prominent sources of lipopolysaccharide, and other pathogen associated molecular patterns (PAMPs) (20, 59, 60), which could conceivably influence m6A regulating pathways in epithelial and immune cells (23, 52, 53). While we cannot infer causality from our cross-sectional design, these findings support the concept of a microbiota-epitranscriptome-immunity axis in CVID.

We did not detect differences in the abundance of selected m6A regulating or RNA-editing enzymes at the protein level between CVID and controls or between CVID subgroups. This does not exclude altered m6A dynamics. Enzyme activity can be modulated by post translational modifications, subcellular localisation and cofactors, and protein levels does not necessarily reflect enzymatic activity. In addition, we only analysed a limited set of m6A-related enzymes and cannot exclude alterations in other relevant enzymes.

The functional consequences of individual m6A marks are highly site and context dependent, determined by the repertoire and activity of reader proteins, which can promote either decay or stabilisation/translation of the same modified transcript (9, 61–63). Consistent with this, we observed only limited overlap between differentially m6A methylated transcripts and proteins with concordant changes, which likely reflects not only limited proteome coverage but also additional layers of post transcriptional and post translational regulation that uncouple mRNA/m6A status from steady state protein abundance, in line with previous large scale integrative studies showing only modest correlations between mRNA and protein levels (11, 64, 65).Our study has several limitations. The sample size is modest, reflecting the rarity of CVID and the challenges of obtaining paired integrated microbiota and m6A (epitranscriptomic) analyses. This limits statistical power, particularly for microbiota-m6A correlation and proteomics integration and increases the risk of both type I and type II errors (5). In addition, the small number of patients with identified monogenic variants and the lack of prospective genotype stratified inclusion may have influenced the analyses and make it difficult to assess the specific impact of monogenic CVID like disorders on m6A regulation. We used bulk tissue, which precludes attribution of m6A changes to specific cell types and may blur signals from less abundant immune populations. Finally, our analyses are correlative and do not address functional consequences of individual m6A marks or their causal role in gut inflammation (9, 66).

Despite these constraints, this exploratory study provides a first glimpse into the epitranscriptomic landscape of the CVID gut. They suggest that m6A mediated regulation of RNA metabolism, particularly in pathways related to mitochondria, ribosomes, protein turnover and RNA silencing, may be involved in shaping local immune responses and their interaction with the mucosal microbiota. Our observations are hypothesis generating and should be interpreted with caution. Future studies should extend and validate these observations using larger cohorts, cell type resolved approaches, higher resolution microbiome profiling and functional assays to dissect how specific m6A marks and their readers influence mucosal immunity in CVID. Such work may ultimately reveal novel targets for modulating chronic gut inflammation in this and related immunodeficiencies.

## Data Availability

The original datasets for the current study are not publicly available due to Norwegian legislation regarding general data protection regulation but are available from the corresponding author (SJ), on reasonable request.
