# Supplementary material for: Altered duodenal N6-methyladenosine levels in common variable immunodeficiency associate with duodenal microbiota
Source: Front Immunol. 2026 Jul 8;17:1875823. doi: 10.3389/fimmu.2026.1875823 (PMC13388135; doi:10.3389/fimmu.2026.1875823)
Supplement: Supplementary file 1 [file DataSheet1.pdf]

Supplementary Information

Altered Duodenal N6-methyladenosine Levels in Common Variable Immunodeficiency Associates with Duodenal Microbiota

Vegard Myhre MSc[1,2], Mari Kaarbø PhD[3], Mingyi Yang PhD[3,4], Børre Fevang MD, PhD[2,5], Mirta M. L. Sousa PhD[6,7], Henrik M. Reims MD, PhD[8], Knut E. A. Lundin MD, PhD[2,9], Johannes R. Hov MD, PhD[1,2,9,10], Pål Aukrust MD, PhD[1,2,5], Magnar Bjørås Cand Scient[3,6], Silje F. Jørgensen MD, PhD[1,5*]

*Corresponding author: s.f.jorgensen@ous-research.no

1. Research Institute of Internal Medicine, Division of Surgery and Specialized Medicine, Oslo University Hospital, Oslo, Norway.
2. Institute of Clinical Medicine, University of Oslo, Oslo, Norway.
3. Department of Microbiology, Oslo University Hospital and University of Oslo, Oslo, Norway.
4. Department of Medical Biochemistry, Oslo University Hospital, Oslo, Norway.
5. Section of Clinical Immunology and Infectious Diseases, Oslo University Hospital, Rikshospitalet, Oslo, Norway.
6. Department of Clinical and Molecular Medicine, Norwegian University of Science and Technology, NTNU, Trondheim, Norway.
7. Proteomics and Modomics Experimental Core Facility (PROMEC) at Norwegian University of Science and Technology, Trondheim, Norway
8. Department of Pathology, Oslo University Hospital, Rikshospitalet, Oslo, Norway
9. Section of Gastroenterology, Department of Transplantation Medicine, Oslo University Hospital, Rikshospitalet, Oslo, Norway
10. Norwegian PSC Research Center, Department of Transplantation Medicine, Oslo University Hospital, Oslo, Norway

Supplementary Information

Supplementary Materials and Methods

Supplementary Figures

Supplementary Figure S1: Unsupervised heatmap of the 4,134 DMTs by condition.

Supplementary Figure S2: Distribution of differentially methylated transcripts by RNA type

Supplementary Figure S3: Alpha diversity in samples

Supplementary Figure S4: Sparse canonical correlation analysis

Supplementary Figure S5: Expression of m6A related enzymes

Supplementary Tables

Supplementary Table S1: Proteins targeted by mass spectrometry to measure their expression

Supplementary Table S2: mRNA differentially methylated transcripts, CVID_N compared to CVID_IEL

Supplementary Table S3: Clinical characteristics for m6A bacteria

Supplementary Table S4: Clinical characteristics for m6A enzymes

Supplementary Table S5: Correlation of significant m6A mRNA transcripts to significant proteins.

Supplementary Excel Files (separate files)

Supplementary Excel File 1: Differentially methylated transcripts

Supplementary Excel File 2: Gene set enrichment analysis results

Supplementary Excel File 3: Microbiota and m6A correlation analysis

Supplementary Excel File 4: m6A enzymes expression

Supplementary Excel File 5: Proteomics and m6A correlation analysis

Supplementary Materials and Methods

Study design

CVID patients were also clinically characterised according to the presence or absence of non-infectious complications, using previously published criteria. Briefly, the Complications subgroup included patients with recurrent bacterial respiratory tract infections and at least one of the following non-infectious complications: splenomegaly, lymphoid hyperplasia, granulomas, chronic diarrhoea, organ specific autoimmunity, autoimmune cytopenia, interstitial lung disease, nodular regenerative hyperplasia in the liver or lymphoma, based on previously defined criteria (1). The Infection only subgroup included patients with recurrent bacterial respiratory infections but none of these non-infectious complications. In this study, "chronic diarrhoea" was defined as diarrhoea lasting more than three months, based on a GI symptom questionnaire (Gastrointestinal Symptom Rating Scale–Irritable Bowel Syndrome, GSRS IBS) and exclusion of GI infection (2).

Microarray analysis

Microarray analysis was performed by Arraystar (Rockville, Maryland, USA) according to their Human m6A-mRNA&lncRNA Epitranscriptomic microarray protocol (https://www.arraystar.com/epitranscriptomic-array-service-m6a/m5c/m1a/ac4c/m7g/-/). Briefly, RNA from each biopsy sample was quantified using NanoDrop ND-1000, and RNA-integrity was assessed by Bioanalyzer 2100 or Mops electrophoresis. RNAs were then immunoprecipitated with an anti-m6A antibody, and m6A-modified RNAs were eluted using magnetic beads and designated as immunoprecipitated, or "IP". The unmodified RNAs were recovered from the supernatant and designated "Sup". The "IP" and "Sup" RNA-fractions were then labelled with Cy5 and Cy3 respectively, as cRNAs in separate reactions using Arraystar RNA labeling protocol. The cRNAs were combined and hybridized onto Arraystar

Human mRNA&lncRNA Epitranscriptomic Microarray (8x60K, Arraystar). The slides were washed and analysed using two-colour channels in an Agilent Scanner G2505C. Agilent Feature Extraction software (version 11.0.1.1) was used to analyse the array images, and the raw intensities of “IP” (immunoprecipitated, Cy5-labelled) and “Sup” (supernatant, Cy3-labelled) were normalized using the average of log2-scaled Spike-in RNA intensities;

$\mathbf{IP}_{Cy5\_normalized\_intensity} = \log2(\mathbf{IP}_{Cy5\_raw}) - \text{Average}[\log2(\mathbf{IP}_{spike\text{-}in\_Cy5\_raw})]$

$\mathbf{Sup}_{Cy3\_normalized\_intensity} = \log2(\mathbf{Sup}_{Cy3raw}) - \text{Average}[\log2(\mathbf{Sup}_{spike\text{-}in\_Cy3\_raw})]$

%Modified was finally calculated by using the ratio of normalized IP (i.e. m6A modified RNA) to total RNA:

$\%\text{Modified} = (\text{modified RNA})/(\text{Total RNA}) = \text{IP}/(\text{IP+Sup}) = \mathbf{IP}_{Cy5\_normalized\_intensity} / (\mathbf{IP}_{Cy5\_normalized\_intensity} + \mathbf{Sup}_{Cy3\_normalized\_intensity})$

There were 39,200 probes in total on the microarray, split into 32,000 mRNAs, 6,316 lncRNAs, 855 pri-miRNA, 497 pre-miRNA, 394 snoRNA, and 18 snRNA.

Data transformation and stabilization

Methylation data was imported into R (v4.2.3) using RStudio (v2023.03.1) with the tidyverse (v2.0.0) and here (v1.0.1) package. The same packages were also used for the other analyses in R for this supplementary. In our analysis we were interested in the percentage of modification per transcript, and we used %Modified from the microarray analysis. These values were a percentage ranging from 0-1, and they were logit-transformed median-normalized by transcript type and by sample.

PCA

PCA was performed using prcomp() from the stats (v3.6.2) package. stat_ellipses() from ggplot2 were used with default settings for calculating and showing a 95% confidence interval for a multivariate t-distribution per condition.

Heatmap and UpSet plot

ComplexHeatmap (v2.14.0) was used for heatmaps and Upset plots. For the heatmap in the main manuscript, the column_split argument was set to the conditions as to cluster the top dendrogram first by condition and mean methylation, and then samples. No column_split argument was used for the heatmap in the supplementary, for an unsupervised clustering of samples.

Moderated t-test

Statistical analyses of methylation were performed using the limma (v3.54.2) package. The normalized data was split by transcript type before fitting models, and moderated t-tests were used for statistical analysis. Limma's arrayWeights() function was used to adjust the model for relative reliability (i.e. homoscedasticity) for each sample. P-values were adjusted for false- discovery rate (FDR) using Benjamini-Hochberg correction. A "CVID_ALL" group was made prior the statistical analysis by combining CVID_N with CVID_IEL.

Enrichment analysis

The packages clusterProfiler (v4.6.2), AnnotationDbi (v1.64.1) and org.Hs.eg.db (v3.18.0) were used for gene set enrichment analysis (GSEA) from the Gene Ontology (GO) database. Ranking lists for GSEA were calculated using –log10(p-value), multiplied by 1 if hypermethylated and by -1 if hypomethylated. The GSEA was run as two-tailed.

m6A enzymes, sample preparation for targeted mass spectrometry

Protein pellets obtained using the AllPrep DNA/RNA/Protein Mini Kit (Qiagen) were resuspended in 8M urea, 4 % CHAPS containing 5 mM TCEP and incubated for 1 hour at room temperature. After measuring protein concentration using the BioRad Bradford assay, 20 µg protein were alkylated with iodoacetamide (1 µmol/mg protein) for 30 min in the dark. Proteins were precipitated using a methanol-chloroform method as described previously and resuspended in 45 µl 50 mM NH4HCO3 containing Trypsin (Thermo Scientific) at a 1:50 ratio (w/w, enzyme:protein) prior to overnight digestion at 37◦C in a shaker. Subsequently, samples were dried in a speed vac and resuspended in 40 µl formic acid followed by centrifugation for 10 minutes at max speed (16 000 g) for removal of insoluble particles prior to mass spectrometry analysis. 20 µl samples were transferred to HPLC vials and a pool of synthetic peptides containing heavy-labelled Lysine (+8) or Arginine (+10) (PEPotec SRM Grade 2 Peptides, Thermo scientific) was spiked into the samples at final concentration 4 fmol/µl.

Targeted mass spectrometry of m6A enzymes

Protein pellets were obtained from snap-frozen pieces of duodenal biopsy samples using the DNA, RNA and protein AllPrep kit (Qiagen, Hilden, Germany), according to the manufacturer's instructions with a few modifications as previously described (3). The protein pellets were resuspended and broken down to peptides by trypsin, followed by separation by liquid chromatography and MS/MS spectrometry.

All parallel reaction monitoring (PRM)-based targeted mass spectrometry methods were designed, analyzed, and processed using Skyline software version 22.2.0.351.8. In silico selection of proteotypic peptides was performed via Skyline using the Homo sapiens reference proteome available at www.uniprot.org to exclude non-unique peptides.

Heavy labelled peptide standards were first analyzed on a Thermo Scientific Q Exactive HF mass spectrometer operating in PRM mode. This data was imported into skyline and used for the selection of the top ionizing peptides (2+ and 3+ charge states) and to build a scheduled method with retention time windows of 7 min. The method was then employed for detection and quantification of corresponding peptides in the samples. Here, information on retention time and fragmentation pattern of the heavy labelled peptide standards was used for peptide identification and chromatographic quality control. The same instrument parameters described below for sample analysis were adopted for establishment of the PRM method with standard peptides.

Tryptic digests (2 µg) together with synthetic heavy peptide standards (20 fmol) were analyzed on a Q Exactive HF mass spectrometer operating in PRM mode coupled to an EASY-nLC 1200 UHPLC system (Thermo Scientific). Peptides were injected onto an Acclaim PepMap C18 column (75 µm i.d. × 2 cm nanoviper, 3 µm particle size, 100 Å pore size) (Thermo Scientific) and further separated on an EASY SprayTM LC column (75 µm i.d. × 50 cm nanoviper, 2 µm particle size, 100 Å pore size) (Thermo Scientific) at 40 oC. The following 120 min method was used at 300 nl/min flow rate: starting with 6 % solvent B (80 % Acetonitrile, 0.1% Formic acid) with an increase to 31 % solvent B in 105 min, followed by an increase to 100 % solvent B over 6 min, where it was subsequently held for 9 min. Solvent A consisted of 0.1% Formic acid. The peptides eluting from the column were ionized by an Easy SprayTM Source (Thermo Scientific) and analysed on positive-ion mode using electrospray voltage 1.75 kV and HCD fragmentation. Each MS/MS scan was acquired at a resolution of 60 000 FWHM, normalized collision energy (NCE) 28, automatic gain control (AGC) target value of 2 × 105, maximum injection time (mIT) of 110 ms and isolation window 1.4 m/z.

Protein Quantification by targeted mass spectrometry

Quantification of peptides detected in the samples was achieved by summing the integrated peak areas of the most intense fragments. Peptide areas for multiple peptides of the same protein were summed to assign relative abundance to that protein. A minimum of 2 peptides per protein was used for quantification. Endogenous ACTB, GAPDH and TUBB levels were used for data normalization using their geometric mean.

Data was imported into R, and missing peptides were imputed using mice (v3.16.0), and the peptides were then summed to the protein level. All proteins were log2-transformed and normalized by subtracting the geometric mean of the expression of ACTB, GAPDH and TUBB (i.e. three housekeeping proteins) for each sample. Statistical analysis was performed using t-tests from the rstatix (v0.7.2) package, with p-values adjusted for FDR using Benjamini-Hochberg correction.

m6A to protein correlation

Proteins were extracted as described in a previous article (3). Briefly, proteins were isolated from snap-frozen pieces of duodenal biopsy samples using protein AllPrep DNA/RNA/Protein Mini Kit (Qiagen, Hilden, Germany) according to the manufacturer's instructions with a few modifications. Precipitated protein pellets were subjected to enzymatic digestion, and the peptides were analysed using liquid chromatography–tandem mass spectrometry platform consisting of an EASY-nLC 1200 ultra-high-performance liquid chromatography system coupled to a Q Exactive HF mass spectrometer operating in FullMS-ddMS2 mode (Thermo Fisher Scientific, Waltham, Mass). The proteins were quantified by processing mass spectrometry (MS) data using MaxQuant (MQ, v1.6.17.0). Protein expressions were imported into R and log2 transformed and normalized. Statistical analysis was performed in the limma package (v3.54.2), using moderated t-tests and arrayWeights() as

already described in “Moderated t-test” above. Our protein data and m6A data was both annotated to the gene level, which resulted in an overlap of 4,101 annotated genes. The m6A data includes mRNA isoforms, and 8,926 mRNAs could therefore be annotated to the 4,101 proteins available. The m6A data included mRNAs for 16,354 different genes in total, which with a proteomic coverage of 4,101, gives a 25% coverage.

Correlation analysis of diversity features and m6A

The microbiota diversity features were imported into R as a csv-file and in centred-log-ratio format. Pairwise Mann-Whitney U tests were used identify differences in microbiota alpha diversities. For m6A values we used the raw, non-median normalized non-logit transformed methylation values, as it is easier to interpret and this correlation analysis investigated the overall degree of methylation, instead of at individual transcripts. m6A values were calculated for each sample using the median of all transcripts, and base R's lm() function from the stats (v3.6.2) package was then used to fit linear models and calculate correlations. glance() from the broom (v1.0.6) package was used for easier extraction of R^2 and p-values.

Four samples from each of CVID_N, CVID_IEL and controls were used in the correlation analysis, based on the availability of matched m6A and microbiota data.

Sparse canonical correlation analysis (sCCA)

Sparse canonical correlation analysis (sCCA) was performed using the PMA (v1.2.4) package.

sCCA was performed in two steps. In the first step we ran CCA.permute() with nperms=800, niter=80 and standardize=FALSE. For sCCA we used the logit-transformed and median normalized methylation values. We set CCA.permute() to iterate over lambdas ranging from 0.1 to 0.7, adding 2/3 in each step for a total of 10 lambdas. These lambdas were used on each dataset, i.e. we iterated over 100 possible combinations of 10 different lambdas from each dataset. We then inspected the permutation test result manually, and chose lambdas based on a combination of high correlation, low p-value, high absolute z-score, and a low number of m6A-transcripts and microbes without overfitting. In the second step we ran CCA() with the chosen lambdas, set with niter=80. We then extracted and visualized the ten highest microbiota loadings from the first canonical variate.

Figures

R-packages ggplot2 (v3.5.0), ggpubr (v0.6.0), ggh4x (v0.2.8), and scales (v1.3.0) were used for figures. ggtext (v1.2.3) was used in some figures for html-formatting of labels and text in plots. RColorBrewer (v1.1.3) was used for palettes with more colour-blind friendly colours. ComplexHeatmap (v.2.14.0) was used for the heatmap and Upset-plots.

Manuscript preparation

The manuscript was written in Rstudio using rmarkdown (v2.30).

Supplementary Figures

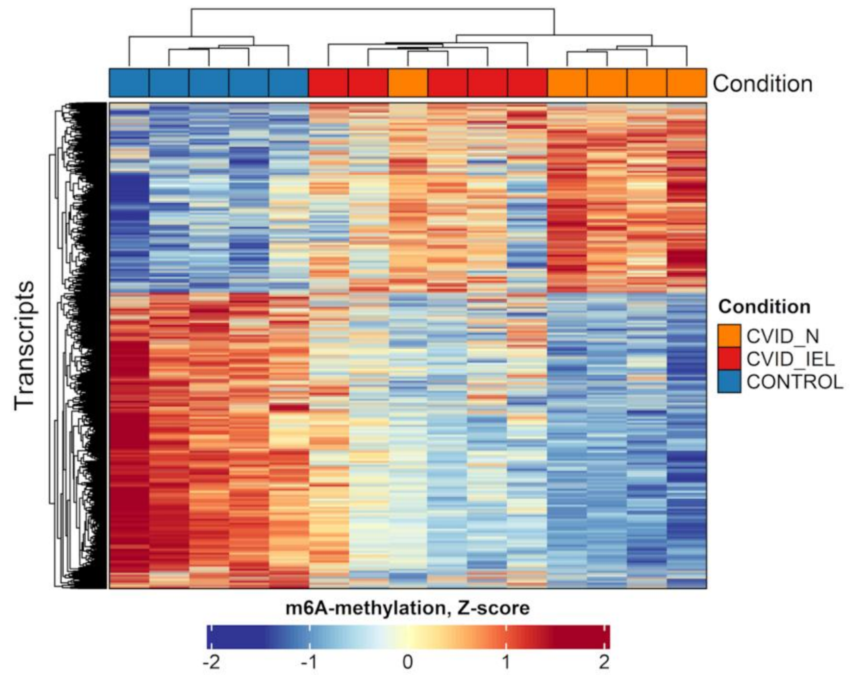

Supplementary Figure S1: Unsupervised heatmap of the 4,134 DMTs by condition. *Top dendrogram show clustering of samples, and left dendrogram show clustering of DMTs. CVID, common variable immunodeficiency; CVID_IEL, CVID with increased intraepithelial lymphocytes and inflammation in the duodenum; CVID_N, CVID patients with normal duodenum.*

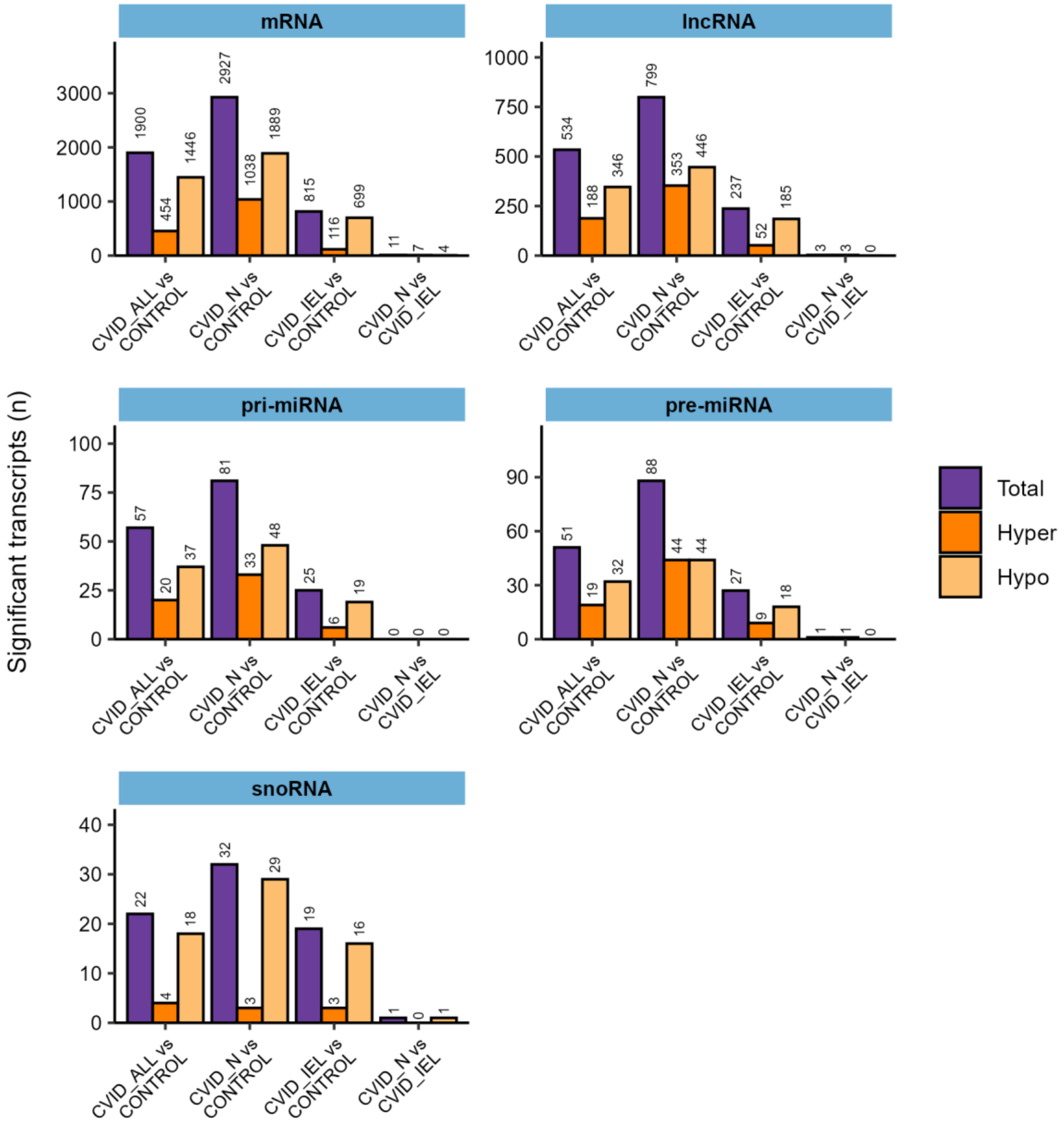

Supplementary Figure S2: Distribution of differently methylated transcripts by RNA type. *Bar plots show the number of differentially methylated transcripts in CVID_ALL (all CVID patients) versus controls, CVID_N (CVID patients with normal duodenal histology) versus controls, CVID_IEL (CVID patients with increased intraepithelial lymphocytes) versus controls, and CVID_N versus CVID_IEL for each RNA class: mRNA, lncRNA, pri miRNA, pre miRNA and snoRNA. For each comparison, bars indicate the total number of differentially methylated transcripts (purple), as well as the number of hypermethylated (orange) and hypomethylated (yellow) transcripts.*

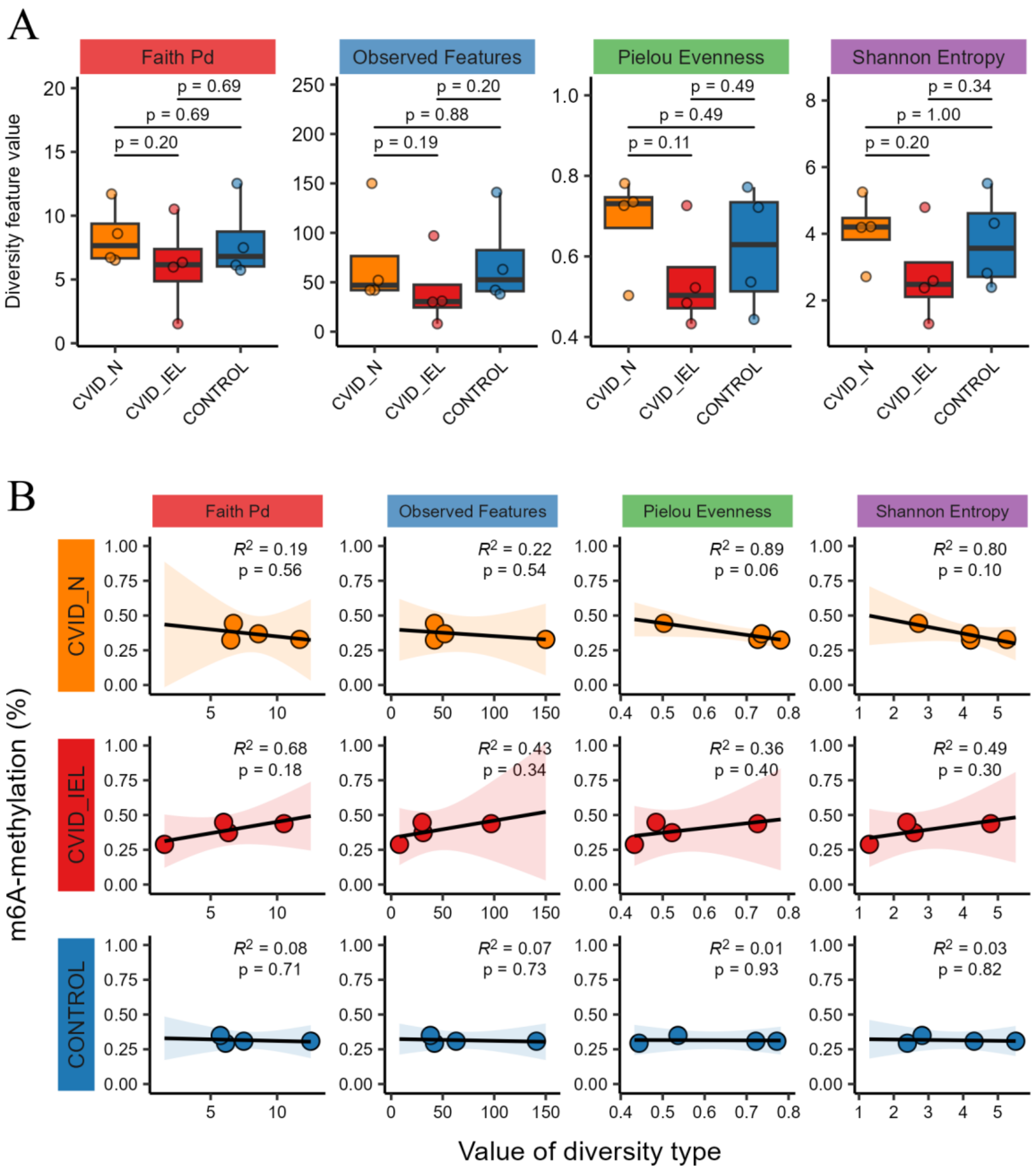

Supplementary Figure S3: Alpha diversity the m6A–microbiota cohort. A) *Alpha diversity metrics (Faith's phylogenetic diversity, observed features, Pielou's evenness and Shannon entropy) in duodenal microbiota from CVID_IEL and CVID_N patients included in the m6A-microbiota correlation cohort. P-values calculated using pairwise Mann-Whitney U test.* ***B)*** *Correlation between these alpha diversity metrics and median m6A methylation across all transcripts at the sample level. No comparisons reached statistical significance (P ≥ 0.05).). CVID, common variable immunodeficiency; CVID_IEL, CVID with increased intraepithelial lymphocytes and inflammation in the duodenum; CVID_N, CVID patients with normal duodenum.*

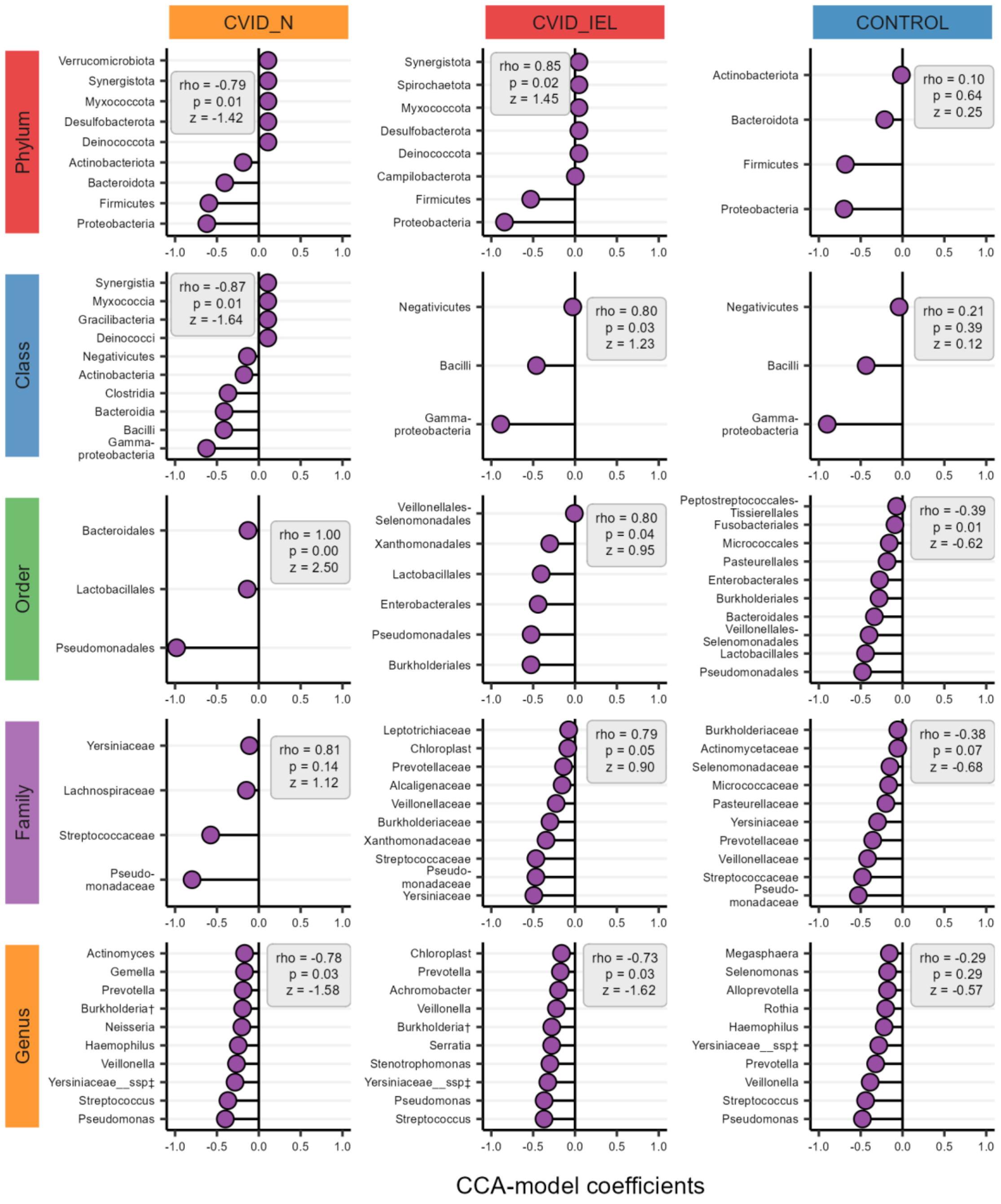

Supplementary Figure S4: Sparse canonical correlation analysis of m6A and microbiota across taxonomic levels. *Top 10 bacterial taxa (phylum, class, order, family and genus) that contribute the most to the sparse canonical correlation models. The higher the absolute value of the coefficients, the greater the impact of the taxa on the model. Rho shows the Pearson correlation for the model. P-values and z-scores were calculated using permutation. CVID, common variable immunodeficiency; CVID_IEL, CVID with increased intraepithelial lymphocytes and inflammation in the duodenum; CVID_N, CVID patients with normal duodenum.*

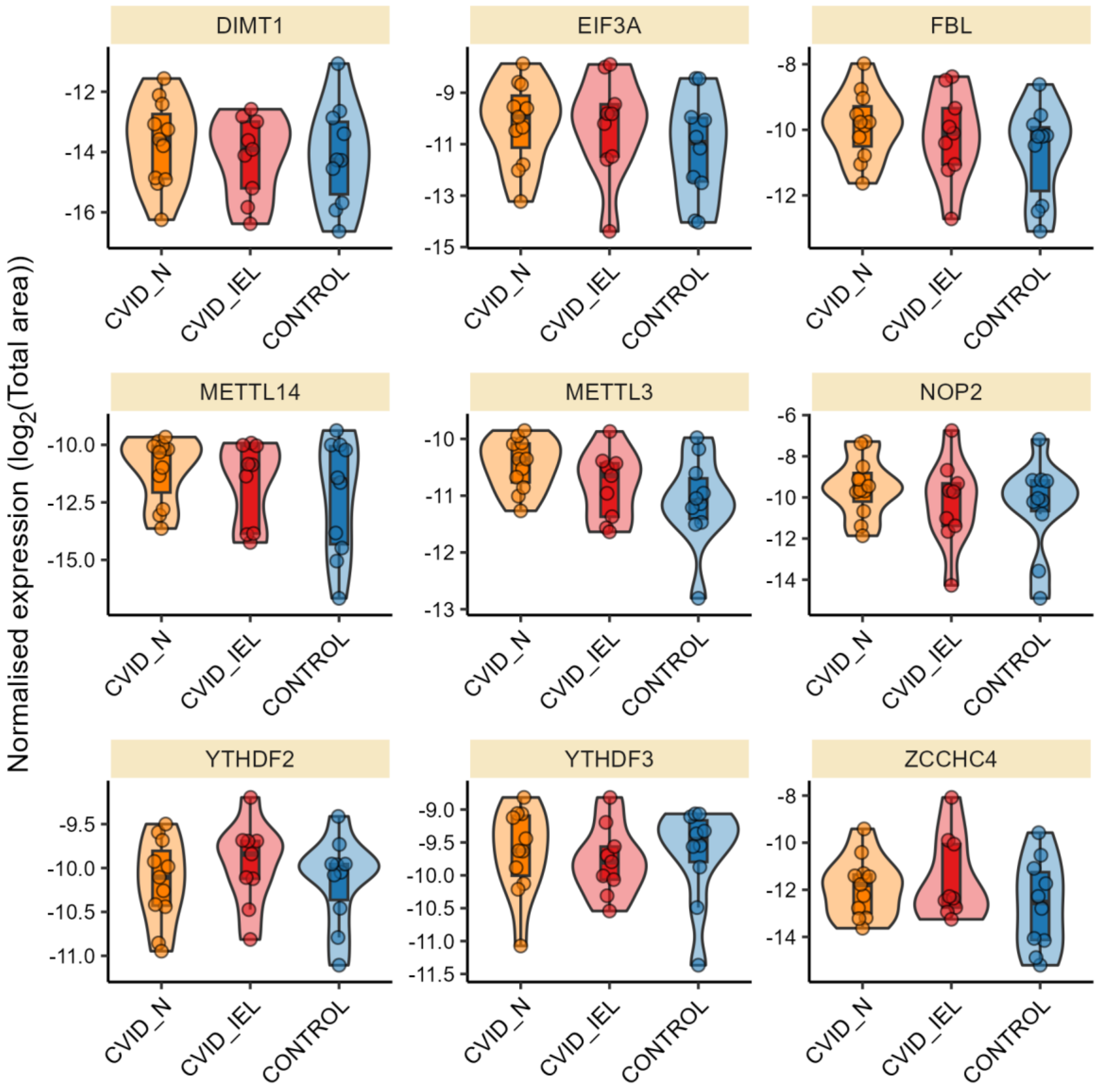

Supplementary figure S5: Expression of m6A-regulating and RNA-editing enzymes in duodenal biopsies. *Expression of nine reliable detected enzymes from a targeted panel of 12 m6A regulating and four RNA editing enzymes in CVID_IEL (n=11), CVID_N (n=9) and controls (n=10), quantified by targeted LC MS/MS. Expression values were normalised to three housekeeping proteins with higher abundance than the m6A-related enzymes, resulting in negative log2 expression values (i.e. log2(enzymes) – log2(housekeeping protein). CVID, common variable immunodeficiency; CVID_IEL, CVID with increased intraepithelial lymphocytes and inflammation in the duodenum; CVID_N, CVID patients with normal duodenum.*

Supplementary Tables

Supplementary Table S1: Proteins targeted by mass spectrometry, including m6A-regulating and RNA-modifying enzymes and housekeeping proteins.

Protein/enzyme	Uniprot entry	Type of enzyme	Detection status
METTL14	Q9HCE5	m6A writer. Mediates N6-methyladenosine (m6A) methylation of RNAs	Detected
METTL3	Q86U44	m6A writer. Mediates N6-methyladenosine (m6A) methylation of RNAs	Detected
METTL5	Q9NRN9	m6A writer. Methylates adenine in 18S rRNA	Not detected
WTAP	Q15007	m6A writer. Mediates N6-methyladenosine (m6A) methylation of RNAs	Not detected
ZCCHC4	Q9H5U6	m6A writer. Methylates adenine in 28S rRNA	Detected
EIF3A	Q14152	m6a reader. Facilitate cap independent mRNA translation.	Detected
YTHDF1	Q9BYJ9	m6A reader. Specifically recognizes and binds N6-methyladenosine (m6A)-containing mRNAs, promoting mRNA degradation	Not detected
YTHDF2	Q9Y5A9	m6A reader. Specifically recognizes and binds N6-methyladenosine (m6A)-containing mRNAs, promoting mRNA degradation	Detected
YTHDF3	Q7Z739	m6A reader. Specifically recognizes and binds N6-methyladenosine (m6A)-containing mRNAs, promoting mRNA degradation	Detected
ALKBH3	Q96Q83	m6A eraser. Demethylates N6-methyladenosine (m6A) RNA	Not detected
ALKBH5	Q6P6C2	m6A eraser. Demethylates N6-methyladenosine (m6A) RNA	Not detected
FTO	Q9C0B1	m6A eraser. Demethylates N6-methyladenosine (m6A) RNA	Not detected
DIMT1	Q9UNQ2	Dimethylates two adjacent adenosines in the loop of a conserved hairpin near the 3'-end of 18S rRNA in the 40S particle	Detected
FBL	P22087	Involved in pre-rRNA processing by catalyzing the site-specific 2'-hydroxyl methylation of ribose moieties in pre-ribosomal RNA	Detected
NOP2	P46087	m5C writer. Specifically methylates 28S rRNA	Detected
NSUN5	Q96P11	m5C writer. Specifically methylates 28S rRNA	Not detected
ACTB	P60709	Housekeeping protein. Used for normalization.	Detected
GAPDH	P04406	Housekeeping protein. Used for normalization.	Detected
TUBB	P07437	Housekeeping protein. Used for normalization.	Detected

Supplementary Table S2: Differentially methylated mRNA transcripts, CVID_N compared to CVID_IEL

Gene	Hypo-/Hyper-methylated	UniProt entry	Function
AGO4	Hyper	Q9HCK5	Required for RNA-mediated gene silencing (RNAi). Binds to short RNAs such as microRNAs (miRNAs) and represses the translation of mRNAs which are complementary to them.
AURKB	Hyper	Q96GD4	Serine/threonine-protein kinase component of the chromosomal passenger complex (CPC), a complex that acts as a key regulator of mitosis.
COQ10B	Hyper	Q9H8M1	Required for the function of coenzyme Q in the respiratory chain. May serve as a chaperone or may be involved in the transport of Q6 from its site of synthesis to the catalytic sites of the respiratory complexes
ETFDH	Hyper	Q16134	Accepts electrons from electron transfer flavoprotein (ETF) and reduces ubiquinone. Component of electron-transfer system in the mitochondria.
ITCH	Hyper	Q96J02	Several functions.<br>Involved in the control of inflammatory signaling pathways (PubMed:19131965).<br>Essential component of a ubiquitin-editing protein complex, comprising also TNFAIP3, TAX1BP1 and RNF11, that ensures the transient nature of inflammatory signaling pathways (PubMed:19131965).
NOM1	Hyper	Q5C9Z4	Plays a role in targeting serine/threonine-protein phosphatase PP1-alpha catalytic subunit (PPP1CA) to the nucleolus
TCF7L2	Hyper	Q9NQB0	Participates in the Wnt signaling pathway and modulates MYC expression by binding to its promoter in a sequence-specific manner. Acts as a repressor in the absence of CTNNB1, and as activator in its presence. Activates transcription from promoters with several copies of the Tcf motif 5'-CCTTTGATC-3' in the presence of CTNNB1. TLE1, TLE2, TLE3 and TLE4 repress transactivation mediated by TCF7L2/TCF4 and CTNNB1. Expression of dominant-negative mutants results in cell-cycle arrest in G1. Necessary for the maintenance of the epithelial stem-cell compartment of the small intestine
CENPJ	Hypo	Q9HC77	Plays an important role in cell division and centrosome function by participating in centriole duplication
SIX1	Hypo	Q15475	Transcription factor that is involved in the regulation of cell proliferation, apoptosis and embryonic development. Regulates the expression of numerous genes, including *MYC*, *CCND1* and *EZR* (By similarity).
TNFAIP8L3	Hypo	Q5GJ75	Acts as a lipid transfer protein. Preferentially captures and shuttles two lipid second messengers, i.e., phosphatidylinositol 4,5- bisphosphate and phosphatidylinositol 3,4,5-trisphosphate and increases their levels in the plasma membrane. Additionally, may also function as a lipid-presenting protein to enhance the activity of the PI3K-AKT and MEK-ERK pathways
ZNF227	Hypo	Q86WZ6	May be involved in transcriptional regulation.

Supplementary Table S3: Clinical characteristics for m6A bacteria

Characteristic	CVID_N	CVID_IEL	Controls	P-value
**Number of patients, n**	4	4	4	
**Female, n**	3	1	2	0.77
**Age (years), mean(min-max)**	44 (28-61)	44 (35-68)	52 (32-67)	0.69
**Infection only**	0	0	-	1.00
**Complications**			-	
Autoimmune cytopenia, n	1	1	-	1.00
Chronic diarrhoea, n*	1	4	-	0.14
Lymphoid-hyperplasia, n	3	3	-	1.00
Organ specific autoimmunity, n	0	1	-	1.00
Splenomegaly, n	2	4	-	0.43
Autoimmunity, n	1	2	-	1.00

CVID_IEL refers to CVID patients with increased intraepithelial lymphocytes and inflammation in the duodenum, while CVID_N refers to CVID patients without this increase and inflammation. P-values were calculated using Fisher's exact, except in age were Kruskal-Wallis was used.

**chronic diarrhoea was defined as diarrhoea lasting more than three months, based on a GI symptom questionnaire (Gastrointestinal Symptom Rating Scale–Irritable Bowel Syndrome, GSRS-IBS) and exclusion of GI infection.*

Supplementary Table S4: Clinical characteristics for m6A enzymes

Characteristic	CVID_N	CVID_IEL	P-value
**Number of patients, n**	11	9	
**Female, n**	7	5	1.00
**Age (years), mean(min-max)**	48 (28-68)	40 (29-58)	0.24
**Infection only**	2	1	1.00
**Complications**	9	8	1.00

CVID_IEL refers to CVID patients with increased intraepithelial lymphocytes and inflammation in the duodenum, while CVID_N refers to CVID patients without this increase and inflammation. P-values were calculated using Fisher's exact, except in age were Kruskal-Wallis was used.

Supplementary Table S5: Overlap between significant m6A-modified mRNA transcripts to significant proteins. The m6A dataset covered approximately 32,000 methylated RNA transcripts, corresponding to 15,354 genes. Of these, methylation data from 8,926 mRNAs could be linked to the protein dataset, which included 4,101 quantified proteins (not a 1:1 ratio due to multiple mRNA isoforms per gene). This corresponds to an overall coverage of roughly 25% of methylated mRNAs at the protein level.

**Comparison**		**CVID_ALL: CONTROL**	**CVID_N: CONTROL**	**CVID_IEL: CONTROL**	**CVID_N: CVID_IEL**
**Number of significant m6A mRNA transcripts in total (n)**	p-adjusted	3,991	4,999	1,101	12
	p-raw	9,323	10,234	6,027	2,911
**Number of significant m6A mRNA transcripts after annotation to protein dataset (n)**	p-adjusted	972	1,313	240	3
	p-raw	2,476	2,852	1,543	893
**Number of significant proteins in total (n)**	p-adjusted	22	13	16	0
	p-raw	446	413	420	239
**Number of proteins that are both significant and has a corresponding significant m6A mRNA transcript (n)**	p-adjusted	5	4	1	0
	p-raw	208	221	130	46
**Percentage of significant proteins that also show significant m6A mRNA transcripts**	p-adjusted	23%	31%	6%	NA
	p-raw	47%	54%	31%	12%

References

1. Chapel H, Lucas M, Lee M, Bjorkander J, Webster D, Grimbacher B, et al. Common variable immunodeficiency disorders: Division into distinct clinical phenotypes. Blood [Internet]. 2008 Jul;112(2):277–86. Available from: https://doi.org/10.1182/blood-2007-11-124545
2. Wiklund I, Fullerton S, Hawkey C, Jones R, Longstreth G, Mayer E, et al. An irritable bowel syndrome-specific symptom questionnaire: Development and validation. Scandinavian journal of gastroenterology. 2003;38(9).
3. Kaarbø M, Yang M, Hov JR, Holm K, de Sousa MML, Macpherson ME, et al. Duodenal inflammation in common variable immunodeficiency has altered transcriptional response to viruses. Journal of Allergy and Clinical Immunology [Internet]. 2023;151(3):767–77. Available from: https://www.sciencedirect.com/science/article/pii/S0091674922013355
